# High-throughput single-cell analysis reveals progressive mitochondrial DNA mosaicism throughout life

**DOI:** 10.1126/sciadv.adi4038

**Published:** 2023-10-25

**Authors:** Angelos Glynos, Lyuba V. Bozhilova, Michele Frison, Stephen Burr, James B. Stewart, Patrick F. Chinnery

**Affiliations:** ^1^ Department of Clinical Neurosciences, School of Clinical Medicine, University of Cambridge, Cambridge Biomedical Campus, Cambridge, UK.; ^2^ Medical Research Council Mitochondrial Biology Unit, University of Cambridge, Cambridge Biomedical Campus, Cambridge, UK.; ^3^ Biosciences Institute, Faculty of Medical Sciences, Wellcome Centre for Mitochondrial Research, Newcastle University, Newcastle upon Tyne, UK.

## Abstract

Heteroplasmic mitochondrial DNA (mtDNA) mutations are a major cause of inherited disease and contribute to common late-onset human disorders. The late onset and clinical progression of mtDNA-associated disease is thought to be due to changing heteroplasmy levels, but it is not known how and when this occurs. Performing high-throughput single-cell genotyping in two mouse models of human mtDNA disease, we saw unanticipated cell-to-cell differences in mtDNA heteroplasmy levels that emerged prenatally and progressively increased throughout life. Proliferating spleen cells and nondividing brain cells had a similar single-cell heteroplasmy variance, implicating mtDNA or organelle turnover as the major force determining cell heteroplasmy levels. The two different mtDNA mutations segregated at different rates with no evidence of selection, consistent with different rates of random genetic drift in vivo, leading to the accumulation of cells with a very high mutation burden at different rates. This provides an explanation for differences in severity seen in human diseases caused by similar mtDNA mutations.

## Teaser

High-throughput single-cell genotyping reveals progressive somatic mosaicism of mtDNA heteroplasmy throughout life.

## INTRODUCTION

Mammalian mitochondrial DNA (mtDNA) includes 37 essential genes required for oxidative phosphorylation (OXPHOS) and the synthesis of adenosine triphosphate (ATP). Unlike nuclear DNA, cells typically contain hundreds to thousands of mtDNA molecules, with the precise number varying across cell types (*1*). Although most of these molecules are identical (homoplasmic), healthy humans carry a mixed population (heteroplasmy), which typically affects <2% of total mtDNA molecules based on bulk-tissue analysis (*2*). Most low-level heteroplasmic mtDNA variants are benign or have no functional effects, but pathogenic mutations can cause cellular dysfunction and cell death when the proportion of mutant molecules (heteroplasmy fraction) exceeds a critical biochemical threshold. This threshold is both mutation- and cell type–specific (*3*). When pathogenic mutations exceed this threshold in a substantial number of cells, rare multisystemic mitochondrial diseases can arise, affecting ~1 in 4300 individuals (*4*). Similar mutations affecting a smaller proportion of cells contribute to common late-onset multifactorial diseases, particularly involving nondividing (postmitotic) tissues as seen in substantia nigra neurons in Parkinson’s disease (*5*). Thus, understanding the dynamic nature defining heteroplasmy distributions has important implications for human health.

Two main mechanisms are thought to explain how cell heteroplasmy levels change in vivo (*6*). In dividing cells, the partitioning of mtDNA molecules during cytokinesis can lead to different mutation levels in the daughter cells. Over time, this can lead to different cell lineages, with different heteroplasmy levels through the process of vegetative segregation. In addition, unlike nuclear DNA, mtDNA is continuously destroyed and replicated, independent of the cell cycle (relaxed replication). The replication and/or destruction of one mtDNA type ahead of another can also change heteroplasmy levels over time and contribute to differences between cells. Although compelling from a theoretical perspective, experimental evidence supporting these mechanisms has been challenging to gather. This is partly due to the limited availability of in vivo animal models, many of which were generated by mixing polymorphic genotypes harboring several different nucleotide substitutions and no single pathogenic mtDNA mutations (*7*–*11*). In addition, our capacity to perform single-cell heteroplasmy measurements at scale has also been limited. Here, we address both of these challenges, studying mice that carry two different pathogenic mtDNA mutations that closely resemble ones found in human patients (*12*, *13*). Our approach involves measuring single-cell heteroplasmy levels from animals at different ages throughout life. Observing extreme variation in heteroplasmy levels at the single-cell level in different organs, we show that the rate of segregation differs between mutations but was near identical in both proliferating and nondividing cells, leading to the accumulation of cells containing a high heteroplasmy level during life. These findings cast light on the mechanisms underpinning heteroplasmy segregation within different cell populations, potentially informing treatment strategies aimed at reducing the mutation burden.

## RESULTS

### High-throughput isolation and heteroplasmy measurement in single cells

**Fig. 1.**
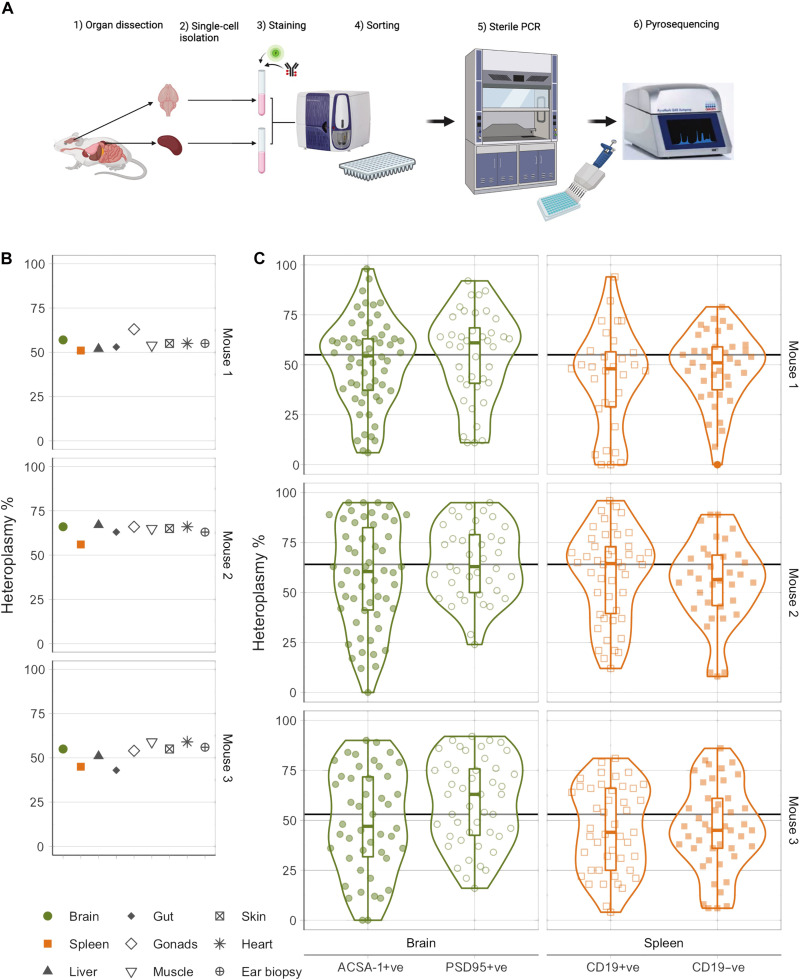
Extreme cell heterogeneity revealed in vivo in the spleen and brain of adult mouse models of mitochondrial disease. (**A**) Single-cell isolation and sequencing pipeline. (**B**) Bulk tissue heteroplasmy measurements from three adult (P100) mice carrying the m.5024C>T mutation. Variability across tissues was small [normalized variance *V*′(*h*) = 0.008 ± 0.005]. (**C**) Single-cell heteroplasmy measurements in different cell types across the brain and spleen. While the average heteroplasmy is stable across tissues, significant variability is observed in each cell population [*V*′(*h*) = 0.203 ± 0.045]. Black line denotes the bulk tissue average for each animal.

We initially studied mice harboring the heteroplasmic m.5024C>T mtDNA mutation in the mitochondrial transfer RNA alanine (tRNA^Ala^ or *mt-Ta*) gene (*12*). m.5024C>T affects tRNA stability leading to a defect of intramitochondrial protein translation and affecting OXPHOS (*12*). It corresponds to the known disease-causing m.5650G>A mutation in humans (*14*, *15*). In addition to DNA extraction from bulk tissue samples, we established a high-throughput tissue-dissociation and fluorescence-activated cell sorting (FACS) pipeline to isolate intact single cells for individual mtDNA genotyping (Fig. 1A). We compared the spleen, a rapidly dividing tissue, to the brain, a largely postmitotic tissue. Mice with a C57BL/6J genetic background were bred under identical conditions in the same facility, and samples were collected immediately after culling (table S1). Spleen-derived immune cell populations were selected as an example of rapidly dividing cells and were FACS sorted into two groups. Mature B lymphocytes (B cells) were identified as CD19+ve, having a lifespan of ~80 days (*16*). The CD19−ve population, encompassed other immune cell types residing within the spleen, including monocytes, granulocytes, dendritic cells, natural killer cells, and macrophages (fig. S1) (*17*). With the exception of T cells, this second group of immune cells has a lifespan of less than 10 days (*16*). Brain cells were selected as an example of quiescent cell types and were also sorted into two groups. In the neonatal mouse brain, astrocytes are a postmitotic cell type with an estimated half-life of 161 days in the mouse hippocampus (*18*). These were isolated on the basis of the presence of surface marker astrocyte cell surface antigen 1 (ACSA-1). By contrast, neural stem and progenitor cells were isolated by being double positive for ACSA-1 and prominin-1 (*19*, *20*). In the case of adult mouse brains, where neural stem and progenitor cells were more limited, postsynaptic density protein 95 (PSD95) was used as an established marker of postsynaptic neurons (fig. S2) (*21*–*23*). The gating strategy was optimized to exclude debris and compromised cells, enabling the isolation of intact DNA-containing cells [Draq5 positive and 4′6-diamidino-2-phenylindole (DAPI) negative], followed by a previously validated pyrosequencing-based single-cell heteroplasmy measurement assay (*13*, *24*), with a mean absolute deviation of 2.8% (fig. S3).

### Extreme mtDNA heteroplasmy at the single-cell level in adult mice

Our initial analysis of 100-day postpartum adult mice (P100) showed low tissue-to-tissue heteroplasmy variability in three m.5024C>T mice (Fig. 1B), as previously demonstrated (*12*, *25*). Next, we studied the distribution of heteroplasmy levels in 122 CD19+ve cells and 118 CD19−ve, 158 ACSA-1+ve, 125 ACSA−1, and PSD95+ve cells. When grouping together cells derived from the same animal (pseudo-bulk), the mean of each single-cell distribution was within 5.16% of the bulk tissue measurement (absolute error of 2.89% ± 2.14%) in keeping with the technical variability of the single-cell genotyping. Despite the similar mean levels between tissues and organs, we saw a large range in single-cell heteroplasmy levels in cell types from both the spleen and the brain [interquartile range (IQR): 40.5 to 75% for the brain and 35.75 to 67% for the spleen; Fig. 1C]. There was no obvious difference in heteroplasmy distribution between different cell types within the same tissue (Kolmogorov-Smirnov, *P* > 0.36 for CD19+ve and CD19−ve cells in the spleen for all three animals and *P* > 0.09 for ACSA-1+ve and ACSA-1/PSD95+ve cells in the brain for all the animals). Different cell populations derived from the same tissue were found to also be comparable at other time points (table S2), and, therefore, the cell populations were combined for each tissue in subsequent analysis.

### Variability in single-cell heteroplasmy levels in dividing and postmitotic tissues with age

**Fig. 2.**
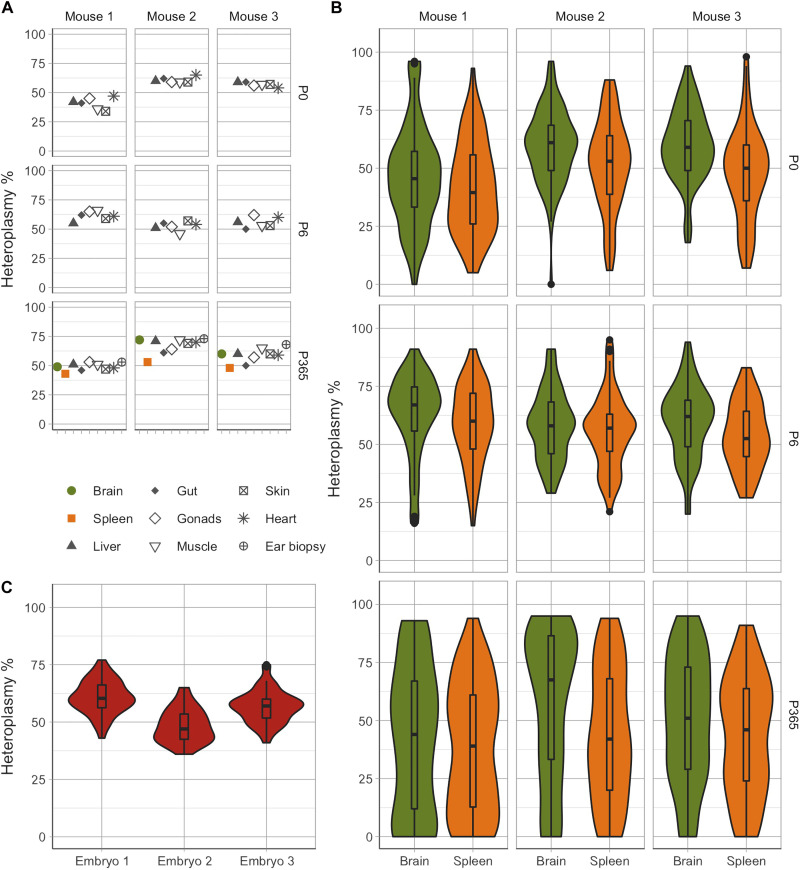
Variability in single-cell heteroplasmy increases with age in the brain and spleen of m.5024C>T mice. (**A**) Bulk tissue heteroplasmy measurements from mice of different ages (P0, P6, and P365) carrying the m.5024C>T mutation. As observed in P100 mice (Fig. 1B), heteroplasmy cross-tissue variability is relatively small at all time points [*V*′(*h*) = 0.007 ± 0.006]. Organ size limitations in P0 and P6 brain and spleen did not allow for bulk tissue measurements to be made. (**B**) Single-cell heteroplasmy measurements in different cell types across the brain and spleen. The variability observed in aged mice (P365) is significantly higher than at earlier time points (*P* = 0.01 for the comparison with E8.5 and *P* < 0.002 for other comparisons, all significant after multiple testing correction). (**C**) Single-cell measurements from random cells of E8.5 embryos carrying the m.5024C>T mutation. E8.5 cell populations exhibited the lowest normalized heteroplasmy variance compared to other time points [*V*′(*h*) = 0.021 ± 0.001].

Next, we explored whether this range of cell heteroplasmy values was present from birth or changed throughout life by performing similar measurements on newborn pups (P0, *n* = 3, *n* = 474 cells across all populations), at 6 days of age (P6, *n* = 3, *n* = 463 cells), and 1-year-old mice (P365, *n* = 3, *n* = 540 cells). Again, the bulk heteroplasmy values across multiple tissues were similar (Fig. 2A), and the range of single-cell heteroplasmy levels was wide for each cell type (Fig. 2B). Despite, on average, having a comparable bulk heteroplasmy levels, we detected only two homoplasmic cells in P0 mice (0.42%), while 49 were identified in P365 mice (9.07%), consistent with ongoing postnatal segregation of mtDNA heteroplasmy throughout adult life.

Heteroplasmy measures are limited by 0 and 100%, rendering most heteroplasmy distributions asymmetric with a variance that depends on the sample mean (*26*, *27*). This means that, even in the absence of selective pressure and subject to identical genetic drift, we expect distributions with extremely high or extremely low mean heteroplasmy to have lower variance compared to distributions with the mean around 50%. To account for this potential confounder, we calculated the normalized heteroplasmy variance (*9*, *28*). This enables a cross-animal comparison, including heteroplasmy distributions of 174 cells obtained from embryonic day 8.5 (E8.5) mice, shortly after organogenesis, to P365 (Fig. 2C). The combined analysis was consistent with an increase in single-cell heteroplasmy variance between prenatal to late adult life, independent of the mean heteroplasmy levels in each mouse and each tissue.

### Mutation-dependent accumulation of homoplasmic cells in dividing and postmitotic tissues

**Fig. 3.**
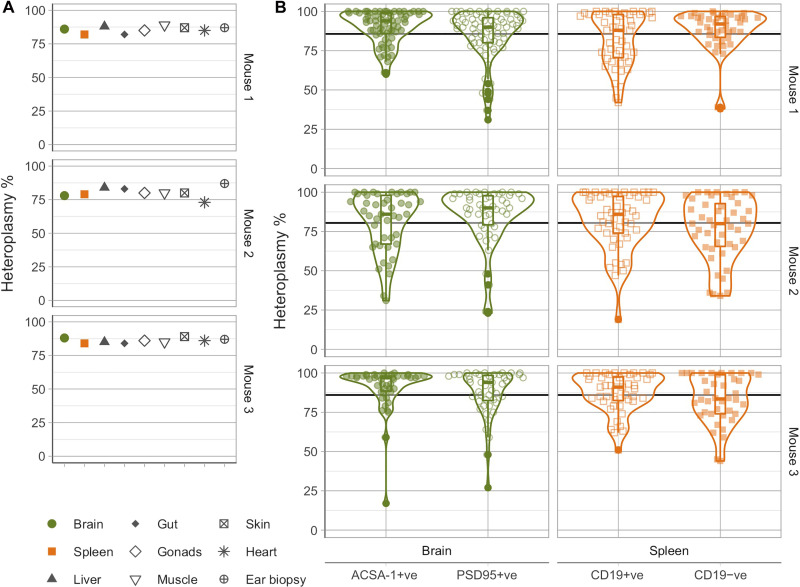
Mutation-dependent accumulation of mutant homoplasmic cells in vivo in the brain and spleen of adult mice carrying the pathogenic m.5019A>G mutation. (**A**) Bulk-tissue heteroplasmy measurements for three mice (P100). Mice carrying the m.5019A>G mutation had higher heteroplasmy compared to m.5024 mice (Fig. 1A) but comparable low variability across tissues. [*V*′(*h*) = 0.006 ± 0.004]. (**B**) Single-cell heteroplasmy measurements in different cell types across the brain and spleen. High variance is observed across all cell populations [*V*′(*h*) = 0.200 ± 0.044], including the accumulation of homoplasmic mutant cells.

**Fig. 4.**
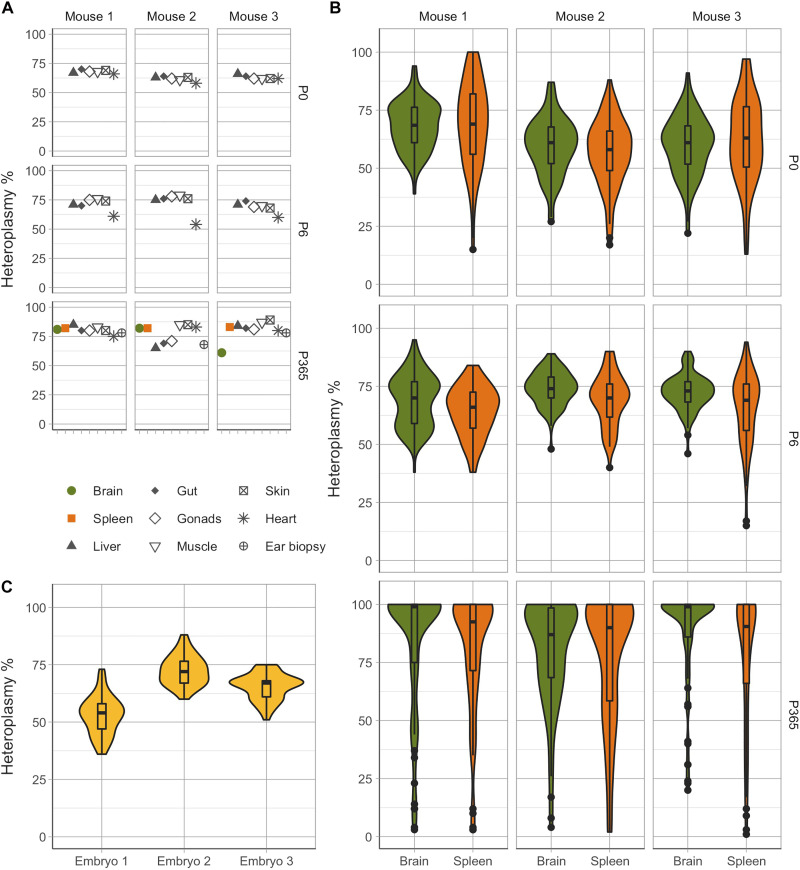
Rapid increase in heteroplasmy variability at the single-cell level leads to the age-dependent accumulation of homoplasmic cells in the brain and spleen of m.5019A>G mice. (**A**) Bulk tissue heteroplasmy measurements from mice of different ages (P0, P6, and P365) carrying the m.5019A>G mutation. As observed in P100 mice (Fig. 3A) and in m.5024C>T mice (Figs. 1B and 2A), heteroplasmy cross-tissue variability is relatively small at all time points [*V*′(*h*) = 0.012 ± 0.016]. Organ size limitations in P0 and P6 brain and spleen did not allow for bulk tissue measurements to be made. (**B**) Single-cell heteroplasmy measurements in different cell types across the brain and spleen. The variability observed in aged mice (P365) is significantly higher than at earlier time points (*P* = 0.012 for the comparison with E8.5 and *P* < 0.002 for other comparisons, all significant after multiple testing correction), with mutant homoplasmic cells observed simultaneously with extremely low-heteroplasmy cells. (**C**) Single-cell measurements from random cells of E8.5 embryos carrying the m.5019A>G mutation. Similarly, to m.5024C>T (Fig. 2C), E8.5 cell populations exhibited the lowest normalized heteroplasmy variance compared to other time points [*V*′(*h*) = 0.021 ± 0.007].

To determine whether this pattern of segregation was mutation-specific, we gathered data from mice carrying the m.5019A>G mutation, which also affects *mt-Ta* but, in contrast to m.5024C>T, does not affect tRNA stability but impairs OXPHOS through a defect on its aminoacylation (*13*). As seen for m.5024C>T, bulk heteroplasmy levels in different tissues were similar in three adult (P100) mice (Fig. 3A), and we observed a wide range of heteroplasmies at the single-cell level in 592 cells across each type (IQR, 81.25 to 98% for the brain and 74 to 97% for the spleen; Fig. 3B). However, unlike m.5024C>T, a high proportion of cells were homoplasmic for m.5019A>G, in keeping with the higher mean heteroplasmy observed in these mice. Extending these observations across the life course, we measured both bulk tissue and single-cell heteroplasmy from P0 (*n* = 3, *N* = 536 cells), P6 (*n* = 3, *N* = 525 cells), and P365 (*n* = 3, *N* = 535 cells) mice (Fig. 4, A and B), as well as single-cell heteroplasmy from E8.5 (*n* = 3, *N* = 141 cells) mouse embryos (Fig. 4C). Again, we observed an increase in variance in the cell heteroplasmy values in older mice and the lowest normalized variance in embryos. Thus, the progressive segregation of mtDNA heteroplasmy in both dividing and nondividing cells is not limited to the m.5024C>T mutation. Last, as with m.5024C>T, the overall heteroplasmy distributions for m.5019A>G were comparable across cells isolated from the spleen and brain.

### Age-dependent accumulation of homoplasmic cells due to random genetic drift

**Fig. 5.**
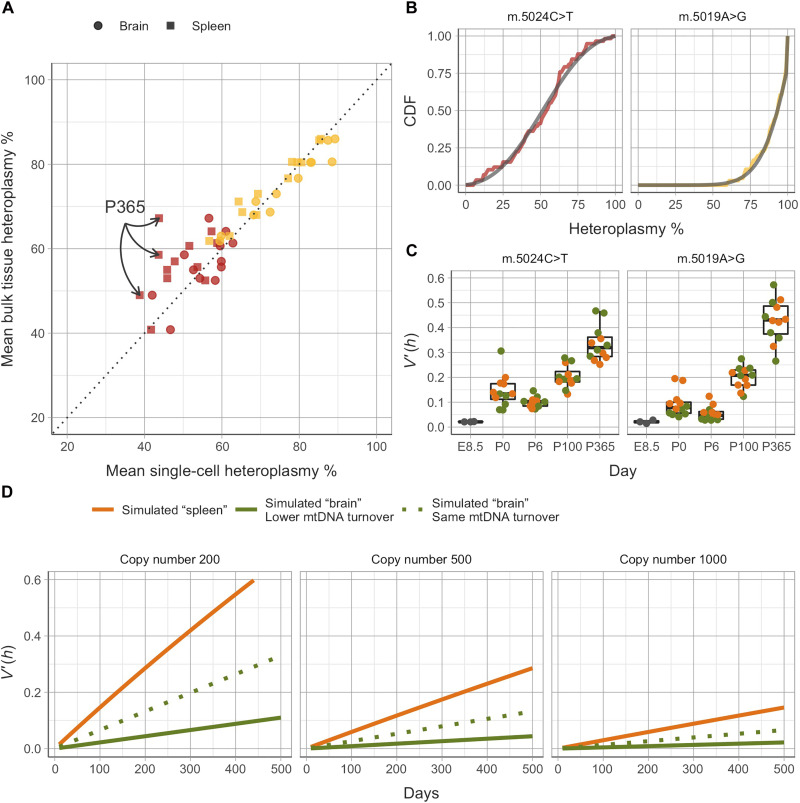
Random genetic drift leads to the age and mutation-dependent accumulation of cells with extreme heteroplasmy values. (**A**) With the exception of the spleen in aged m.5024C>T mice (P365, red squares, highlighted), the mean heteroplasmy in single-cell populations closely matches bulk measurements aggregated over multiple tissues. (**B**) Heteroplasmy of both m.5024C>T and m.5019A>G mutations closely follow the two-parameter Kimura distribution and are therefore consistent with random genetic drift (representative data from P100 shown, see tables S3 and S4). The accumulation of homoplasmic cells at later time points is therefore consistent with genetic drift and not indicative of selection. (**C**) Single cell–normalized heteroplasmy variance *V*′(*h*) is lowest for E8.5 embryos and increases monotonically after P6. Note that *V*′(*h*) is related to the drift parameter of the Kimura distribution. An increase in *V*′(*h*) over time is consistent with existing models of genetic drift (*17*). (**D**) Model of normalized heteroplasmy variance *V*′(*h*) (*17*). Given constant mean copy number, the model predicts that *V*′(*h*) should increase linearly over time due to relaxed replication and vegetative segregation. Quiescent cells (simulated “‘brain,” green dotted line) are therefore predicted to be less variable than proliferating cells (simulated “spleen,” orange line) as they are only subject to relaxed replication and not vegetative segregation. Moreover, quiescent cells have slower mtDNA turnover than proliferating ones (*20*–*23*), resulting in even lower predicted variance (simulated brain, green solid line). This is in contradiction with observed experimental data. Lower copy number and/or higher mtDNA turnover may explain the observed higher variance in spleen cells. Kimura fits and normalized variances were analyzed separately for each single-cell population, without merging at the tissue level.

To explore the mechanism underlying the observed segregation, we first compared the measured bulk heteroplasmy measurements to the calculated mean single-cell heteroplasmy value in each tissue for both mutations (or “pseudobulk”) (Fig. 5A). There was strong correlation (Pearson’s ρ = 0.906) between the measured bulk heteroplasmy level and the pseudobulk value, indicating that the isolated cells were representative of the whole organ and compatible with a mechanism predominantly driven by random genetic drift. The only exception was observed in P365 m.5024C>T spleen cells, which, on average, had lower mean single-cell heteroplasmy levels than the mean bulk. This is in keeping with previous observations that the mutation is selected against when it exceeds 60% heteroplasmy in rapidly dividing tissues such as blood or colonic epithelium in mice at comparable time points (*12*).

To further investigate whether the shapes of the single-cell heteroplasmy distributions were consistent with random genetic drift, the data were fitted to the two-parameter Kimura distribution (*27*). The Kimura distribution models the heteroplasmy fractions of a cell population, which has arisen from a single progenitor with heteroplasmy *p*_0_ and which has undergone genetic drift described by a segregation parameter *b*. When data are fitted to the Kimura distribution, *p*_0_ is typically estimated by the mean heteroplasmy $\hat{p}=\bar{h}$ and $\hat{b}=1-V^{\prime }(h)$. The single-cell heteroplasmy values for both m.5024C>T and m.5019A>G closely matched a Kimura distribution (Fig. 5B and tables S1 and S2). Thus, although the m.5019A>G data appear skewed toward mutant homoplasmy (Figs. 3B and 4B), the observed single-cell heteroplasmy levels were consistent with random genetic drift (*27*) and showed no evidence of selective forces actively favoring the mutant haplotype.

Although we observed a general trend toward an increased heteroplasmy variance from P6 onward (Fig. 5C), the rate of increase was different between m.5024C>T and m.5019A>G mice. From P6 to P365, the m.5024C>T normalized variance increased 3.4-fold from 0.103 (±0.019) to 0.333 (±0.060), while m.5019A>G normalized variance increased 7.8-fold from 0.055 (±0.029) to 0.430 (±0.072). In keeping with this, a linear model incorporating data from P6 and later stages confirmed that the mutation type was a significant predictor of the normalized variance (*P* = 6.97 × 10^−5^ for the interaction term between day and mutation; table S5).

### Importance of relaxed replication in determining cell-to-cell heteroplasmy variability

Next, we compared the heteroplasmy variance between the different tissues. There was no difference in the variance of single-cell heteroplasmy values for the spleen and brain for both m.5024C>T and m.5019A>G mice across time points (Fig. 5C, Wilcoxon’s paired rank test, *P* = 0.967). In keeping with this, tissue type was not a significant predictor of the normalized heteroplasmy variance in a generalized linear model incorporating all variables (*P* = 0.80). These findings were a surprise and contradict what was expected from accepted models describing cell heteroplasmy levels, where the variance in cell heteroplasmy levels is predicted to increase more rapidly in rapidly proliferating spleen cells when compared to nondividing (postmitotic) cells found in the brain. We used established mathematical modeling (*28*) to explore the possible explanations for this apparent paradox.

Assuming a constant mean copy number, existing models of mtDNA dynamics describe heteroplasmy variance increasing linearly with time in the absence of selection, regardless of the exact copy number control mechanism (*28*). This increase is predicted to happen through two processes—relaxed replication and vegetative segregation (see Introduction). Tissues that divide more rapidly and experience faster mitochondrial turnover (such as the spleen) are therefore be expected to have a higher heteroplasmy variance compared to nondividing tissues (such as the brain), particularly in old age. Under these assumptions, normalized heteroplasmy variance *V′*(*h*) can be estimated by the “turnover-adjusted” Wright formula (*29*)

$${\hat{V}}^{\prime }(h)=1-{\left(1-\frac{1}{2n}\right)}^{g}+\frac{4t}{3n\tau }$$

where *n* is the mean copy number per cell at the point of cell division (assumed to be constant over the lifetime of the mouse), *g* is the number of cell divisions, and τ is the mtDNA turnover rate, ${\left(1-\frac{1}{2n}\right)}^{g}$ is the term associated with cell division, and $\frac{4t}{3n\tau }$ is the term associated with relaxed replication.

We measured mtDNA content in representative cell types (fig. S4) and used published estimates for the cell division rates in the spleen (*17*) and mtDNA turnover in the blood and postmitotic tissues (*30*–*33*) to compare the predicted increase in variance for the spleen and brain to our experimental observations (Fig. 5D). Using a slower mtDNA turnover rates in nondividing compared to proliferating cells (half-life of 21 and 7 days, respectively) (*20*–*23*), the model predicted a greater than sixfold difference in the normalized heteroplasmy variance in the spleen than the brain at P365. This was not consistent with the observed experimental data (Fig. 5C), indicating that either the model and/or the assumed parameters were incorrect. To explore the importance of the underlying parameters, we assumed the mtDNA turnover rates to be equal in both cell types (half-life of 7 days). Despite this adjustment, there was still a more than twofold difference in the predicted normalized heteroplasmy between the spleen and brain at P365 when compared to P0 (Fig. 5D). In this context, the difference between the predicted and observed variance could only reflect cell division in spleen cells (*g* > 0) but not brain cells (*g* = 0). These findings were replicated across different mean copy number and mtDNA turnover rates. In conclusion, the experimental findings in the brain indicate that mtDNA turnover independent of the cell cycle is sufficient to cause the range of heteroplasmy values we observed in single cells during life, and the modeling is consistent with the possibility that mtDNA turnover rates in the brain are notably greater than currently thought, potentially exceeding the turnover in the spleen.

## DISCUSSION

Measuring mtDNA heteroplasmy in ~4500 single cells, here, we show that the variance in single-cell heteroplasmy levels increases during life in two different animal models of mtDNA disease. Although predicted by mathematical models (*28*, *34*), to date, there has been limited evidence supporting this in vivo. For both mutations and across both tissues tested, single-cell heteroplasmy levels fitted two-parameter Kimura distribution and are therefore compatible with a mechanism largely governed by random genetic drift. The wide range of heteroplasmy levels we observed was notable, and although, in some cases, the bulk heteroplasmy levels were below the biochemical threshold for each animal, many individual cells exceeded the level known to cause mitochondrial dysfunction. In humans with mtDNA disease, small changes in proportion of high heteroplasmy cells can contribute to disease progression (*35*), prompting the suggestion that a progressive increase in heteroplasmy variance could be deleterious (*28*). Our observations directly support this hypothesis.

Our second finding is that the heteroplasmy variance was the same for rapidly dividing spleen cells and nondividing brain cells. This shows that, in vivo, relaxed replication is likely to be a powerful mechanism determining cell heteroplasmy levels in the absence of cell division. This also raises the possibility that intracellular mtDNA turnover is a more powerful determinant of cell heteroplasmy levels than vegetative segregation. This is important because it provides an explanation for clinical progression of mtDNA disease, which typically involves nondividing tissues.

It is intriguing that at P365 we saw a greater heteroplasmy variance for m.5019A>G than m.5024 T>C. Both mutations affect *mt-Ta*, are 5 base pairs apart, but have subtly different molecular mechanisms. m.5019A>G affects tRNA charging, likely due to disruption of G:U pairing in the acceptor stem (*13*), whereas m.5024C>T compromises the stability of mt-Ta (*12*). The m.5024C>T mutation induces a stronger nuclear transcriptional response than m.5019A>G during embryonic development, and it is challenging to breed mice with m.5024C>T heteroplasmy levels exceeding 80% (*12*). This suggests that m.5024C>T is under greater constraint than m.5019A>G, possibly explaining why the rate of drift is faster for m.5019A>G. We can only speculate about the molecular basis for this, but impaired mitochondrial protein synthesis is one possibility, reducing mtDNA turnover in m.5024C>T mice. In keeping with this, high levels of the m.5024C>T mutation have been shown to affect the translation of mtDNA encoded proteins in heart samples (*12*), but this was not the case for either heart or liver samples carrying the m.5019A>G (*13*).

The decrease in normalized heteroplasmy variance observed in 1-week-old mice (P6) compared to neonates (P0) was an unexpected finding. It is unlikely that this is an experimental artifact because the same trend was observed in all cell types isolated from both tissues in triplicate for both *mt-Ta* mutations. The decrease in heteroplasmy variance immediately after P6 implies selection, but given that the distribution of heteroplasmy levels fitted Kimura distributions at all time points and that the mean single-cell heteroplasmy level matched the bulk measurement, any selection would need to have acted symmetrically on both very high and very low levels. This has been proposed before for m.5024C>T during oocyte maturation (*25*), where a replicative advantage may be counterbalanced by purifying selection. Evidence from mouse cardiomyocytes suggests of a strong *PINK1*, and *Mfn2*-dependent mitophagic clearance of embryonic mitochondria precedes their replacement with new, mature organelles (*36*). While the removal of embryonic mitochondria could explain the slight drop in heteroplasmy variance at P6, cardiomyocytes have also been reported to undergo a 13-fold increase in mtDNA copy number during the first 4 weeks of life (*37*). These responses might not be as prominent in every cell type but provide some context behind the drop (P6) and subsequent increase (P100) in single-cell heteroplasmy variance observed in both *mt-Ta* mouse models. Last, a reduction in cell-to-cell heteroplasmy variance would also arise if there were sharing of mtDNA between cells. This could occur through cellular bridges, which form between oocytes in several species (*38*), but have yet to be described during fertilization and P6 in mice.

In humans with mtDNA diseases, the proportion of cells with a biochemical OXPHOS defect correlates with the clinical severity (*39*). This has led to various strategies aimed at reducing the proportion of OXPHOS-deficient cells, including through the induction of mitochondrial biogenesis (*40*, *41*) or increase in mtDNA copy number (*42*). Our observations raise the counterintuitive suggestion that decreasing rather than increasing mtDNA turnover will prevent clinical progression by slowing down the rate of genetic drift. It is conceivable that this could even stop mtDNA mutations reaching levels that cause disease. This hypothesis will require careful validation for other mtDNA mutations and in other species but may actually be a more tractable therapeutic approach in the long term, as inhibiting biological processes with small molecules is generally easier than increasing them. Likewise, anything that accelerates random drift—such as transient mtDNA depletion causing a “bottleneck effect”—is likely to increase the number OXPHOS-deficient cells at a younger age. This has been proposed as an explanation for the late complications of anti-HIV drugs, potentially accelerating biological aging (*43*). Thus, understanding how heteroplasmy changes during life has far-reaching implications, and manipulating mtDNA turnover may have unanticipated adverse consequences.

Note that we have only studied two mtDNA mutations in the same gene, *mt-Ta.* While other studies have focused on the transmission of *mt-Ta* mutations down the maternal germ line (*12*, *25*, *44*), we have sought to examine its segregation in somatic tissues. We had previously demonstrated that germline selection acts in a heteroplasmy-dependent manner for the m.5024C>T mutation, limiting the distribution of offspring to between 18 and 83% heteroplasmy (*25*). These same selection limits appear to be missing in the somatic tissues studied here. Recognizing that mt-tRNA mutations are not subjected to the same stringent purifying selection forces that apply to protein-coding ones (*45*), it will be important to examine the behavior of other heteroplasmic mtDNA mutations as and when they are generated. Moreover, expanding our observations to include other examples of both postmitotic (e.g., cardiomyocytes) and rapidly dividing (e.g., colonic epithelium) cell types could further corroborate our findings. Future technologies allowing us to pair single-cell mtDNA copy number to heteroplasmy without impairing the accuracy of the latter could reveal the presence of compensatory responses. Although it will be important to validate these findings in other model systems, the limited human high-throughput single-cell data available at present do support our findings (*46*). Critically, our work has shown the importance of in vivo work in developing models to understand mtDNA dynamics and not just relying on cell culture systems, which may be misleading by placing greater emphasis on vegetative segregation than relaxed replication. New in vivo heteroplasmic mouse models provide an opportunity to validate and extend our findings, providing an experimental system to comprehensively dissect out the underlying molecular mechanisms.

## MATERIALS AND METHODS

### Animal husbandry

Two mouse lines were used in this study, the m.5019A>G (allele symbol: mt-Ta^m2Jbst^, Mouse Genome Informatics ID: 6860509) and m.5024C>T (allele symbol: mt-Ta^m1Jbst^, Mouse Genome Informatics ID: 5902095). Mice used as part of this study were bred on the C57BL/6J background. Both lines were bred in Cambridge, United Kingdom, following the directions of the Animal (Scientific Procedures) Act 1986 under the Home Office project license: P6C97520A. All experiments carried out under this license acquired approval by the University of Cambridge Animal Welfare Ethical Review Body and followed the Animal Research Reporting of In Vivo Experiments guidelines. Mice had ad libitum access to water and SAFE 105 universal diet (Safe Diets) while being housed in individually ventilated cages (Tecniplast) and maintained at 20° to 24°C and 45 to 65% humidity. For the collection of embryos at E8.5, breeding females aged 8 to 12 weeks were introduced to stud males after said males were allowed to acclimatize in an empty cage. Checks were then carried out for vaginal plugs each morning and noon. The day when the vaginal plug was detected was designated as E0.5. Culling of animals, including pregnant females for the collection of embryos, was carried out using cervical dislocation methods described in schedule 1 of the Animals (Scientific Procedures) Act of 1986 and confirming the procedure’s success by observing permanent cessation of circulation.

### Tissue collection and dissociation

Upon culling the animals, embryos or tissues including the brain, ear, heart, gut, spleen, liver, skeletal muscle, gonads, and embryos were dissected and placed within 1.5-ml Eppendorf tubes containing Leibovitz’s L-15 medium (Sigma-Aldrich) + 10% fetal bovine serum and then immediately on ice. Spleen dissociation was carried out by cutting the organ into two pieces and placing it in a 6-cm tissue culture dish containing phosphate-buffered saline. Mechanical dissociation followed using the flat-ended thumb rest of a syringe plunger. The remaining debris and cell clumps were removed by filtering the cell suspension through a CellTrics 50-μm cell strainer. Subsequently, the cells were spun down at 300*g* for 10 min at 4°C, and the supernatant was discarded. Red blood cells were then removed from the cell pellet through the use of Miltenyi Biotech Red Blood Cell Lysis Solution. Following a second centrifugation at 300*g* for 10 min at 4°C, the resulting cell pellet was resuspended in 1 ml of phosphate-buffered saline and was placed on ice in preparation for staining. Brain dissociation in animals in the first week of postnatal life was carried out following the protocol outlined in neural tissue dissociation kit (P) provided by Miltenyi Biotech, while, for older mice, the adult brain dissociation kit for mouse and rat, also provided by Miltenyi Biotech, was used. In the case of the adult brain dissociation kit for mouse and rat, slight modifications were applied to the protocol to account for the lack of the gentleMACS Octo Dissociator machine. Instead of following the recommended 37-ABDK-01, three short spins were carried out followed by 10- to 15-min incubations on the basis of the ones described in the neural tissue dissociation kit (P) protocol.

### Fluorescence-activated cell sorting

FACS was conducted on the BD FACS Melody Cell Sorter platform, and analysis of the data was performed using FlowJo software. All samples were kept on ice, while those destined for sorting were stained with Draq5 and DAPI to allow for identification of intact, DNA-containing cells, which take up Draq5 but exclude DAPI. In addition, neonate mouse brain samples were stained with Prominin-1–phycoerythrin (PE)–Vio770 [Miltenyi Biotech, product number (PN): 130-102-153] and GLAST (ACSA-1)–PE (Miltenyi Biotech, PN: 130-118-344), while adult mouse brain samples were stained with GLAST (ACSA-1)–PE (Miltenyi Biotech, PN: 130-118-344) and PSD95 (Abcam, PN: ab18258) used alongside the secondary antibody Briliant Violet 421 (BioLegend, 406410). Mouse spleen samples were stained with CD19–fluorescein isothiocyanate (Abcam, PN: ab86904). All antibodies were used in a 1:200 concentration (v/v) and allowed to incubate for 45 min at 4°C.

### Single-cell pyrosequencing

Single-cell heteroplasmy measurements were carried out as described in (*13*).

### Single-cell digital droplet polymerase chain reaction

mtDNA copy number was measured at the single-cell level using digital droplet polymerase chain reaction (PCR), as described in (*24*).

### Statistical analysis

Normalized heteroplasmy variance for a cell population with heteroplasmies *h* = (*h*_1_, *h*_2_, …, *h_n_*) was calculated as

$$V^{\prime }(h)=\mathrm{var}(h)/\bar{h}(1-\bar{h})$$

This normalization is necessary as the sample variance *var*(*h*) depends on the mean heteroplasmy $\bar{h}$, and heteroplasmy distributions with mean around 50% are expected to have higher variance. The normalization ensures that the variances are corrected for mean heteroplasmy and therefore comparable across animals. Single-cell heteroplasmy distributions were compared using Kolmogorov-Smirnov hypothesis tests. Normalized heteroplasmy variances were compared using Wilcoxon’s rank tests. Goodness-of-fit tests for the Kimura distribution were carried out as described (*25*) using a Monte Carlo Kolmogorov-Smirnov test with *M* = 1000 Monte Carlo simulations. Copy number comparison between m.5024C>T and m.5019A>G ACSA-1 and PROM-positive cells was carried out with a two-sided Wilcoxon’s rank test. In all hypothesis tests, a significance level α = 0.05 was used, and Bonferroni multiple testing correction was applied as appropriate.

The linear model for normalized variance *V*′(*h*) ~ Mutation + Day + Mutation × Day was chosen by forward and backward stepwise Akaike information criterion (AIC) selection, bounded between the null model *V*′(*h*) ~ 1 and the complete model, incorporating mutation, day, tissue, and all possible pairwise interaction terms between these variables. Estimates of the normalized variance using the turnover-adjusted Wright’s formula were carried out with the following parameters based on experimental measurements and the literature (*30*–*33*):

1) Copy number between 100 and 2000, at intervals of 100

2) Time (in days) between 10 and 400, at intervals of 10

3) Cell division rate for proliferating tissue (spleen) of 3 days

4) mtDNA half-life in proliferating cells of 7 days

5) mtDNA half-life in quiescent cells of 7 and 21 days
