# Supplementary material for: High-throughput single-cell analysis reveals progressive mitochondrial DNA mosaicism throughout life
Source: Sci Adv. 2023 Oct 25;9(43):eadi4038. doi: 10.1126/sciadv.adi4038 (PMC10599618; doi:10.1126/sciadv.adi4038)
Supplement: Supplementary file 1 — Figs. S1 to S4 Tables S1 to S5 [file sciadv.adi4038_sm.pdf]

Science Advances
AAAS

Supplementary Materials for

High-throughput single-cell analysis reveals progressive mitochondrial DNA mosaicism throughout life

Angelos Glynos *et al.*

Corresponding author: James B. Stewart, jim.stewart@newcastle.ac.uk

Patrick F. Chinnery, pfc25@cam.ac.uk

Sci. Adv. **9**, eadi4038 (2023)
DOI: 10.1126/sciadv.adi4038

This PDF file includes:

Figs. S1 to S4
Tables S1 to S5

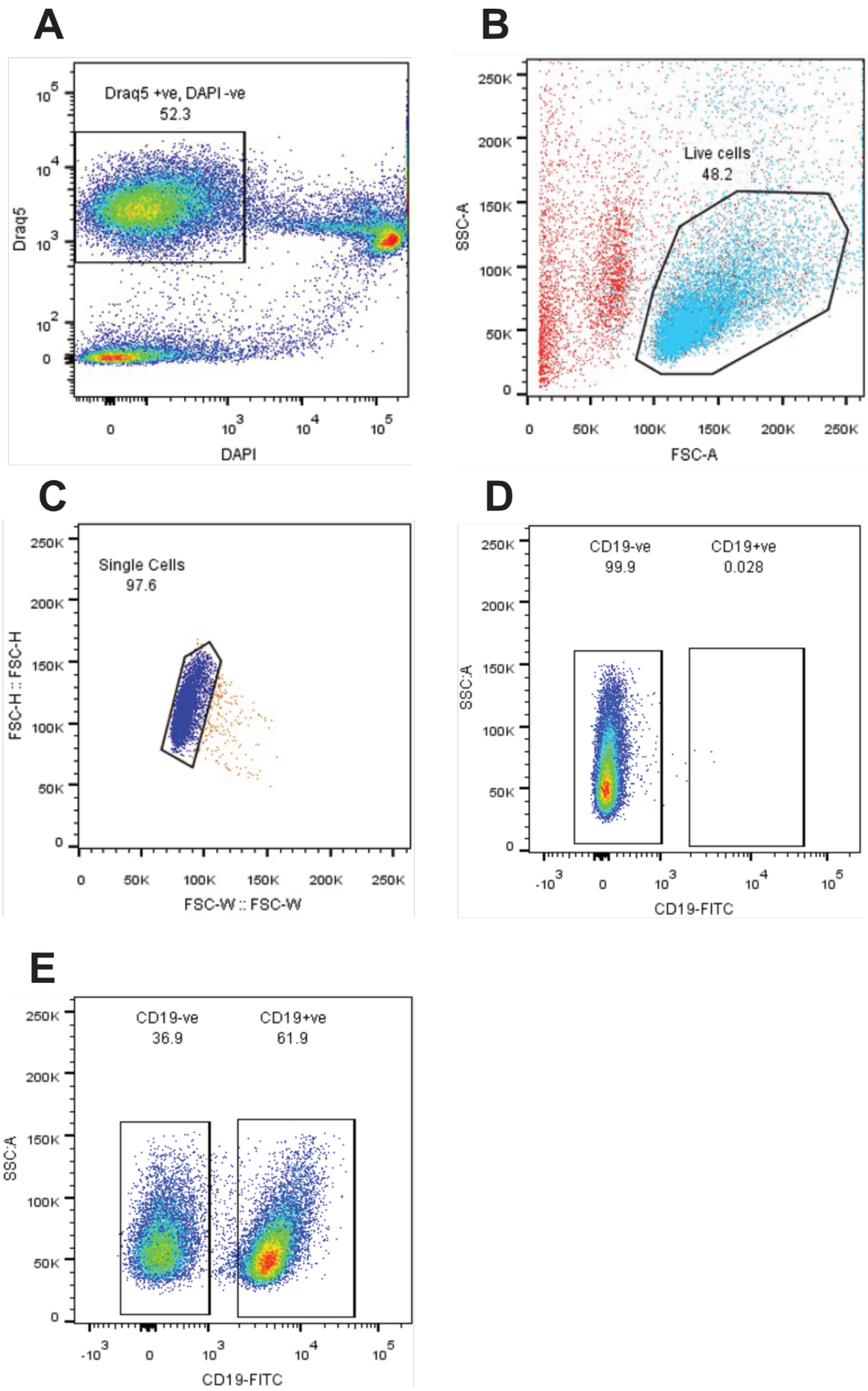
A
Draq5 +ve, DAPI -ve
52.3
Draq5
DAPI
B
Live cells
48.2
SSC-A
FSC-A
C
Single Cells
97.6
FSC-H :: FSC-H
FSC-W :: FSC-W
D
CD19-ve
99.9
CD19+ve
0.028
SSC-A
CD19-FITC
E
CD19-ve
36.9
CD19+ve
61.9
SSC-A
CD19-FITC

Fig. S1.

Isolation of mouse spleen cell populations through FACS. (A) Identification of intact, DNA-containing cells derived from mouse spleen through the application of DRAQ5^{TM} and DAPI staining. **(B)** Determining the forward and side scatter parameters of intact, DNA-containing cells (live cells). **(C)** Doublet exclusion. **(D)** Negative control for FITC fluorescence. **(E)** Discrimination of cell populations based on the expression of CD19-FITC.

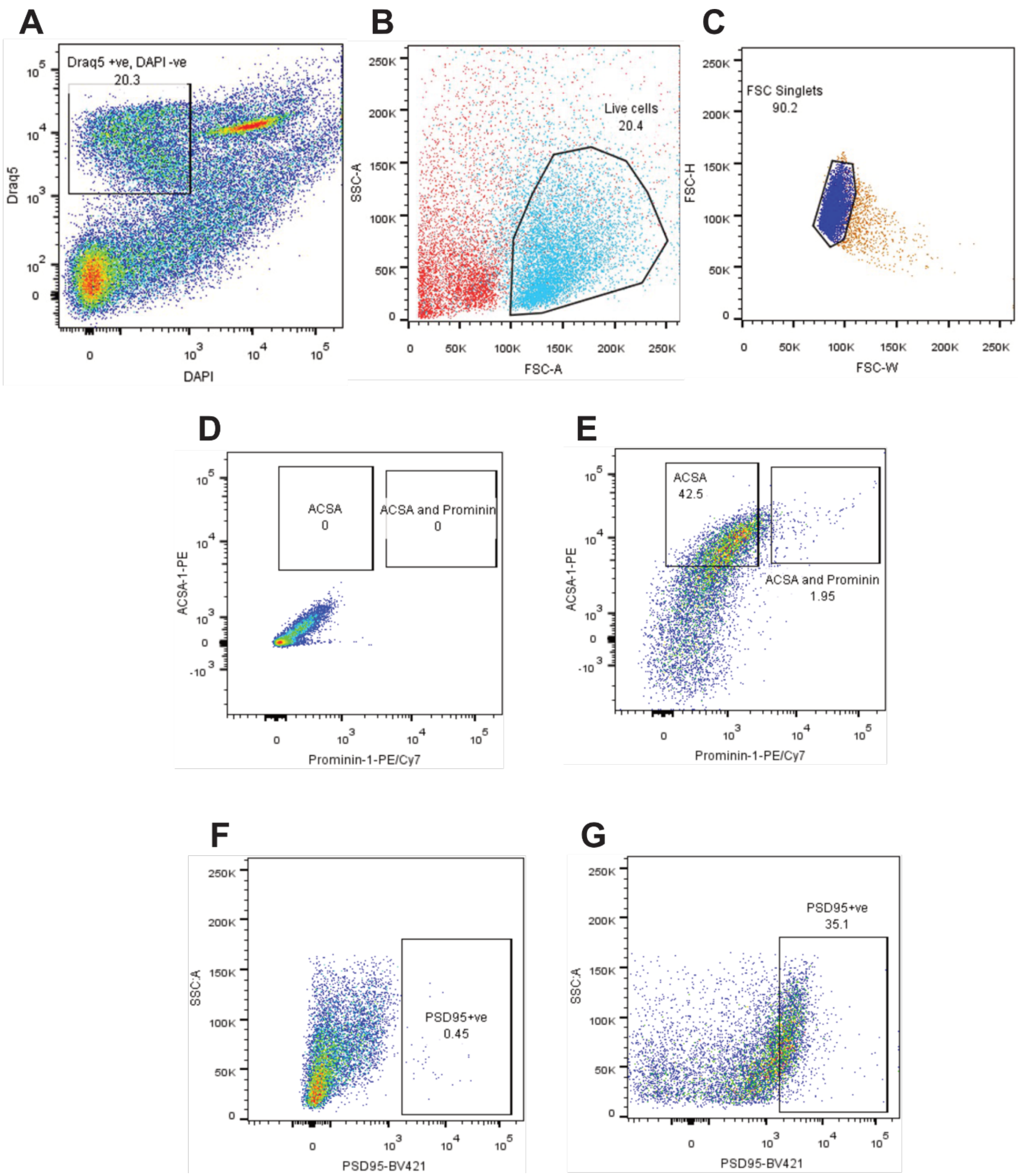

Fig. S2.

Isolation of mouse brain cell populations through FACS. (A) Identification of intact, DNA-containing cells derived from mouse brain through the application of DRAQ5^{TM} and DAPI staining. **(B)** Determining the forward and side scatter parameters of intact, DNA-containing cells (live cells). **(C)** Doublet exclusion. **(D)** Negative control for PE and PE/Cy5

fluorescence. **(E)** Discrimination of target cell populations based on the expression of ACSA-1-PE and Prominin-1-PE/Cy7. **(F)** Negative control for BV421 fluorescence. **(G)** Discrimination of target cell populations based on the expression of PSD95-BV421.

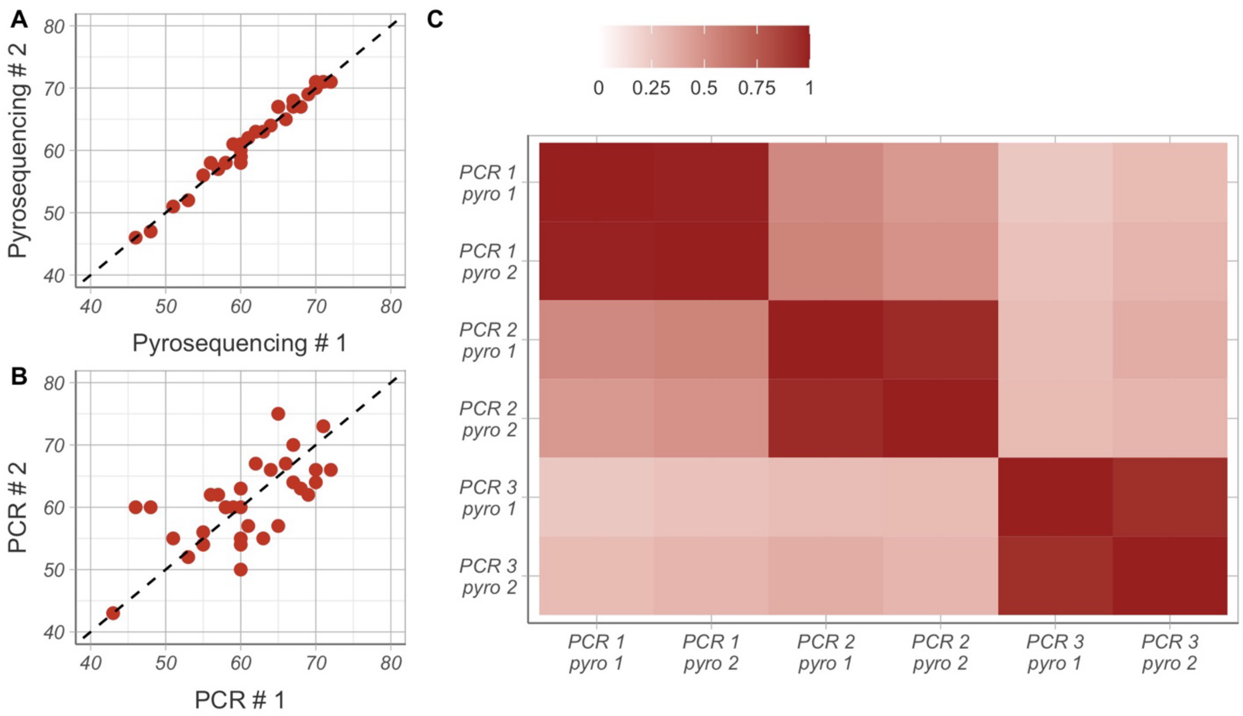

Fig. S3.

Repeat single-cell heteroplasmy measurements (n= 32cells obtained from an E8.5 m.5024C>T mouse embryo). (A) Pyrosequencing replicates of the same PCR sample. **(B)** PCR replicates of the same single-cell lysate. **(C)** Pearson correlation coefficients across replicates. Independent pyrosequencing replicates of the same PCR product were very highly correlated ($\rho > 0.94$ in all cases), while there was more variability across PCR replicates ($\rho \approx 0.50$ between the first two runs PCR 1 and PCR 2, and $\rho \approx 0.30$ between these and PCR 3).

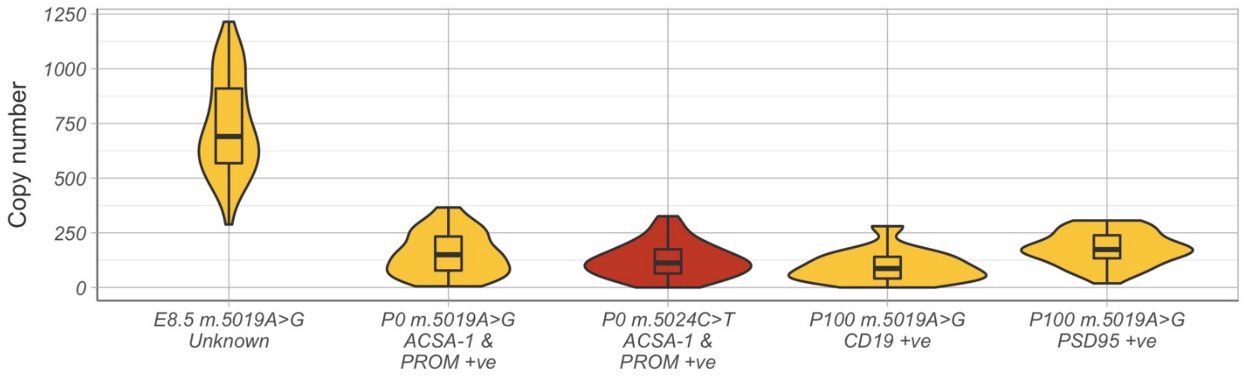

Fig. S4.

Copy number measurements on representative cells. Higher copy numbers were observed in cells obtained from an E8.5 embryo (740±221) compared to cells isolated *post partum* (138±86). ACSA-1 and PROM-+ve cells from m.5024C>T and m.5019>A>G animals were comparable (Wilcoxon's rank test p= 0.21). Cell populations isolated from m.5019A>G mice are shown in yellow, ones isolated from m.5024C>T cells are shown in red.

	Age	Mouse Number	Sex
m.5024C>T	P0	1	M
		2	F
		3	M
	P6	1	M
		2	M
		3	F
	P100	1	M
		2	M
		3	M
	P365	1	M
		2	F
		3	F
m.5019A>G	P0	1	F
		2	M
		3	F
	P6	1	M
		2	M
		3	M
	P100	1	M
		2	M
		3	M
	P365	1	M
		2	F
		3	F

Table S1.
Mouse sex. Defining the sex for all postnatal mice based on the expression of the Y-linked *SRY* gene.

	Day	Mouse number	Tissue	# of cells	KS p-value
m.5024C>T	E8.5	1	unknown	79	NA
		2	unknown	47	NA
		3	unknown	48	NA
	P0	1	brain	84	0.215
			spleen	78	0.741
		2	brain	88	0.267
			spleen	64	0.148
		3	brain	91	0.054
			spleen	69	0.967
	P6	1	brain	72	0.645
			**spleen**	**81**	**0.007**
		2	brain	96	0.12
			spleen	73	0.945
		3	brain	85	0.068
			spleen	56	0.173
	P100	1	brain	98	0.309
			spleen	70	0.485
		2	brain	93	0.095
			spleen	80	0.367
		3	brain	92	0.309
			spleen	90	0.793
	P365	1	brain	89	0.323
			spleen	92	0.634
		2	brain	90	0.166
			spleen	88	0.289
		3	brain	89	0.389
			spleen	92	0.316
m.5019A>G	E8.5	1	unknown	47	NA
		2	unknown	47	NA
		3	unknown	47	NA
	P0	1	brain	92	0.157
			spleen	90	0.105
		2	brain	94	0.762
			spleen	81	0.946
		3	brain	92	0.305
			spleen	87	0.059
	P6	1	brain	115	0.091
			spleen	66	0.996
		2	brain	85	0.452
			spleen	92	0.417
		3	brain	86	0.761
			spleen	81	0.116
	P100	1	brain	138	0.255
			**spleen**	**89**	**0.028**
		2	brain	91	0.457
			spleen	94	0.535
		3	brain	93	0.226
			spleen	87	0.239
	P365	1	brain	81	0.855
			spleen	92	0.111
		2	brain	91	0.191
			spleen	91	0.105
		3	brain	90	0.824
			spleen	90	0.355

Table S2.

Number of cells obtained from each animal. With the exception of E8.5, where random cells were collected, two distinct cell populations were obtained per tissue per animal (CD19 +ve and CD19 -ve cells for spleen; ACSA-1 +ve and double ACSA1 +ve and Prominin-1 or PSD95 +ve for brain). In each case, the two populations within each tissue were compared using a Kolmogorov-Smirnov test. The only two cases where p-values lower than 0.05 were found, are highlighted (m.5024C>T P6 Mouse 1 Spleen p = 0.007, and m.5019A>G P100 Mouse 1 Spleen p = 0.028), they were not significant after multiple testing correction.

Day	Mouse	Tissue	Cell type	$\hat{p}$	$\hat{b}$	p-value
E8.5	Embryo 1	N/A	N/A	0.608	0.978	0.661
	Embryo 2	N/A	N/A	0.484	0.979	0.667
	Embryo 3	N/A	N/A	0.567	0.979	0.783
P0	Mouse 1	Brain	ACSA-1 & PROM +ve	0.468	0.908	0.961
			ACSA-1 +ve	0.466	0.694	0.788
		Spleen	CD19 -ve	0.411	0.801	0.819
			CD19 +ve	0.421	0.866	0.909
	Mouse 2	Brain	ACSA-1 & PROM +ve	0.617	0.93	0.866
			ACSA-1 +ve	0.571	0.867	0.959
		Spleen	CD19 -ve	0.548	0.882	0.433
			CD19 +ve	0.483	0.826	0.805
	Mouse 3	Brain	ACSA-1 & PROM +ve	0.579	0.931	0.705
			ACSA-1 +ve	0.618	0.874	0.749
		Spleen	CD19 -ve	0.48	0.823	0.629
			CD19 +ve	0.476	0.86	0.727
P6	Mouse 1	Brain	ACSA-1 & PROM +ve	0.648	0.879	0.43
			ACSA-1 +ve	0.609	0.854	0.366
		Spleen	CD19 -ve	0.529	0.895	0.958
			CD19 +ve	0.642	0.899	0.926
	Mouse 2	Brain	ACSA-1 & PROM +ve	0.608	0.927	0.529
			ACSA-1 +ve	0.557	0.897	0.529
		Spleen	CD19 -ve	0.549	0.906	0.954
			CD19 +ve	0.566	0.891	0.67
	Mouse 3	Brain	ACSA-1 & PROM +ve	0.552	0.916	0.364
			ACSA-1 +ve	0.636	0.898	0.995
		Spleen	CD19 -ve	0.508	0.926	0.975
			CD19 +ve	0.56	0.915	0.963
P100	Mouse 1	Brain	ACSA-1 +ve	0.513	0.808	0.844
			PSD95 +ve	0.546	0.799	0.423
		Spleen	CD19 -ve	0.482	0.867	0.683
			CD19 +ve	0.429	0.74	0.352
	Mouse 2	Brain	ACSA-1 +ve	0.58	0.722	0.891
			PSD95 +ve	0.652	0.853	0.948
		Spleen	CD19 -ve	0.554	0.823	0.748
			CD19 +ve	0.587	0.789	0.567
	Mouse 3	Brain	ACSA-1 +ve	0.493	0.733	0.959
			PSD95 +ve	0.593	0.806	0.959
		Spleen	CD19 -ve	0.461	0.813	0.748
			CD19 +ve	0.456	0.817	0.74
P365	Mouse 1	Brain	ACSA-1 +ve	0.39	0.533	0.849
			PSD95 +ve	0.451	0.671	0.924
		Spleen	CD19 -ve	0.419	0.702	0.756
			CD19 +ve	0.356	0.661	0.825
	Mouse 2	Brain	ACSA-1 +ve	0.588	0.541	0.239
			PSD95 +ve	0.546	0.621	0.603
		Spleen	CD19 -ve	0.472	0.644	0.918
			CD19 +ve	0.402	0.72	0.997
	Mouse 3	Brain	ACSA-1 +ve	0.519	0.715	0.95
			PSD95 +ve	0.487	0.69	0.977
		Spleen	CD19 -ve	0.454	0.732	0.26
			CD19 +ve	0.419	0.748	0.965

Table S3.

Kimura analysis for m.5024C>T samples. All m.5024C>T single-cell populations fitted the Kimura distribution.

Day	Mouse	Tissue	Cell type	$\hat{p}$	$\hat{b}$	p-value
E8.5	Embryo 1	N/A	N/A	0.53	0.972	0.944
	Embryo 2	N/A	N/A	0.725	0.979	0.857
	Embryo 3	N/A	N/A	0.654	0.986	0.454
P0	Mouse 1	Brain	ACSA-1 & PROM +ve	0.693	0.959	0.613
			ACSA-1 +ve	0.67	0.943	0.915
		Spleen	CD19 -ve	0.652	0.89	0.996
			CD19 +ve	0.724	0.805	0.983
	Mouse 2	Brain	ACSA-1 & PROM +ve	0.586	0.947	0.95
			ACSA-1 +ve	0.603	0.927	0.624
		Spleen	CD19 -ve	0.569	0.927	0.98
			CD19 +ve	0.565	0.906	0.887
	Mouse 3	Brain	ACSA-1 & PROM +ve	0.584	0.95	0.806
			ACSA-1 +ve	0.612	0.917	0.657
		Spleen	CD19 -ve	0.595	0.813	0.915
			CD19 +ve	0.641	0.904	0.944
P6	Mouse 1	Brain	ACSA-1 & PROM +ve	0.704	0.956	0.739
			ACSA-1 +ve	0.673	0.93	0.37
		Spleen	CD19 -ve	0.643	0.958	0.788
			CD19 +ve	0.643	0.942	0.958
	Mouse 2	Brain	ACSA-1 & PROM +ve	0.742	0.97	0.686
			ACSA-1 +ve	0.739	0.972	0.632
		Spleen	CD19 -ve	0.712	0.95	0.889
			CD19 +ve	0.678	0.941	0.947
	Mouse 3	Brain	ACSA-1 & PROM +ve	0.725	0.972	0.793
			ACSA-1 +ve	0.724	0.968	0.769
		Spleen	CD19 -ve	0.662	0.876	0.849
			CD19 +ve	0.643	0.908	0.167
P100	Mouse 1	Brain	ACSA-1 +ve	0.901	0.877	1
			PSD95 +ve	0.85	0.793	0.945
		Spleen	CD19 -ve	0.878	0.831	0.328
			CD19 +ve	0.827	0.807	0.951
	Mouse 2	Brain	ACSA-1 +ve	0.814	0.765	0.983
			PSD95 +ve	0.848	0.725	0.401
		Spleen	CD19 -ve	0.768	0.781	0.999
			CD19 +ve	0.821	0.772	1
	Mouse 3	Brain	ACSA-1 +ve	0.908	0.763	0.001
			PSD95 +ve	0.877	0.791	0.603
		Spleen	CD19 -ve	0.834	0.832	0.83
			CD19 +ve	0.874	0.864	0.985
P365	Mouse 1	Brain	ACSA-1 +ve	0.812	0.428	0.998
			PSD95 +ve	0.847	0.499	0.954
		Spleen	CD19 -ve	0.755	0.518	0.99
			CD19 +ve	0.857	0.675	0.991
	Mouse 2	Brain	ACSA-1 +ve	0.825	0.734	0.781
			PSD95 +ve	0.77	0.641	0.99
		Spleen	CD19 -ve	0.804	0.566	0.988
			CD19 +ve	0.737	0.572	0.922
	Mouse 3	Brain	ACSA-1 +ve	0.884	0.556	0.82
			PSD95 +ve	0.886	0.62	0.883
		Spleen	CD19 -ve	0.761	0.488	1
			CD19 +ve	0.8	0.578	0.94

Table S4.

Kimura analysis for m.5019A>G samples. All m.5019A>G single-cell populations fitted the Kimura distribution. The Kimura test for one population, ACSA-1 +ve brain cells from the P365 Mouse3, had a p-value p= 0.0009, indicating a possible poor fit. This was not significant after multiple testing correction.

	Estimate	Std. Error	t value	Pr (>$\|t\|$)
**(Intercept)**	1.148×10^{-1}	1.370×10^{-2}	8.380	4.47×10^{-12}
**Mutation**	-4.427×10^{-2}	1.938×10^{-2}	-2.285	0.0255
**Day**	6.220×10^{-4}	6.269×10^{-5}	9.921	7.53×10^{-15}
**Mutation ×Day**	3.757×10^{-4}	8.866×10^{-5}	4.237	6.97×10^{-5}

Table S5.
Linear model of the normalized heteroplasmy variance. The best model fit *V '(h)* ~ *Mutation* + *Day* + *Mutation* × *Day* was obtained from backward and forward stepwise model selection by AIC. Tissue and pairwise tissue interaction terms were also considered as variables in the model but were discarded during model selection.
